# Draft genome sequence of emerging pathogen *Morganella morganii* strain PS00513 isolated from a chronic surgical wound

**DOI:** 10.1128/mra.01277-24

**Published:** 2025-03-05

**Authors:** Kira N. Allison, Rayhane Mejlaoui, Katrina G. DeZeeuw, Jonah E. Marek, Edana Cassol, Joerg Overhage

**Affiliations:** 1 Department of Health Sciences, Carleton University 6339 , Ottawa, Ontario, Canada; 2 Department of Complex Continuing Care, Saint Vincent Hospital 6335 , Ottawa, Ontario, Canada; Loyola University Chicago, Chicago, Illinois, USA

**Keywords:** chronic wound, surgical wound, Oxford Nanopore sequencing, antimicrobial resistance, biofilms

## Abstract

We report the draft genome sequence and antimicrobial resistance gene profile of *Morganella morganii* strain PS00513. This strain was isolated from a chronic surgical wound infection of an adult male patient. The chromosome is 3.87 Mb long and accompanied by a 9.6 kb plasmid, which both contain multiple antibiotic resistance genes.

## ANNOUNCEMENT

Chronic wounds are persistent, non-healing wounds often associated with underlying infections and biofilm formation, both of which contribute to delayed wound healing (1). Traditionally, the gram-negative facultative anaerobic gut bacterium, *Morganella morganii*, is becoming increasingly recognized as an emerging opportunistic pathogen in a variety of different infections, including chronic wounds, where its antibiotic resistance and toxin production are concerning factors (2–4).

*M. morganii* strain PS00513 was isolated from a culture swab taken from the edge of a surgical chronic wound on the shin of an adult male patient in Ottawa, Canada. The isolate was selected from Cetrimide Agar (Sigma-Aldrich) supplemented with 0.5 mg/L ciprofloxacin after 37°C incubation for 48 h. For whole genome sequencing, cells were grown at 37°C for 18 h at 280 rpm in Luria–Bertani media. DNA was extracted using the PureLink Microbiome DNA Purification Kit (Invitrogen) following the protocol optimized for microbial culture extraction [MAN0014332 Rev.A (10 Sept. 2015)]. The sequencing library was prepared for Oxford Nanopore Technologies (ONT) using Rapid Sequencing Kit V14 (SQK-RAD114, ONT) and sequenced with MinION (Mk1B) and Flongle R10.4.1 (FLO-FLG114) for 24 h. Read quality was assessed with MinKNOW.v.23.04.3 (q score > 9), and basecaller dorado.v.7.3.9 (https://github.com/nanoporetech/dorado) detected and removed adapter/primer sequences concurrently. High-accuracy mode Guppy.v.6.5.7 (https://nanoporetech.com/) generated fastq files for flye.v.2.9.3-b1797 (5) to perform *de novo* genome assembly (no genome rotation) and overlap detection and trimming. Variant calling, error correction, and polishing were performed with medaka.v.1.11.3 (https://github.com/nanoporetech/medaka) and genome annotation with prokka.v.1.14.5 (6). ResFinder.v.2.4.0 (7, 8) and EPI2ME antimicrobial resistance (AMR) workflow (v2023.04.26-1808834) with input reads aligned against reference sequences in the Comprehensive Antimicrobial Resistance Database (CARD) (9) were used for taxonomic and AMR gene identification. Default parameters and protocols were utilized, unless stated otherwise. Ethical approval for the study was granted by the Carleton University Ethics Committee (118036).

Overall, 130.23 K raw sequencing reads with a mean read length of 7211 kb were used to construct both *M. morganii* chromosome (3,872,624 bp; 51% G + C) and plasmid (9,635 bp; 63.5% G + C). Genome completeness was 80.01%, with 112× coverage, estimated read N50 of 11.77 Kb, and assembly N50 of 3,872,624 bp, resulting in 3,794 predicted genes. The high G + C content of 51% is characteristic of *M. morganii* strains (10, 11) often used to distinguish *Morganella* from other *Proteeae* species (12). Using ResFinder, antibiotic resistance to five antibiotic classes was identified: aminoglycosides (strB_M28829, aph(3′)-Ia_V00359), beta-lactams (blaDHA-17_KM087850, blaTEM-1B_AY458016), folate pathway antagonists (sul1_U12338, dfrA7_AB161450), tetracyclines (tetC_AJ517790), and amphenicol (catA1_V00622). EPI2ME AMR detection found the same resistance phenotypes in addition to 143 alignments to the CARD protein variant model (93.6% average alignment accuracy) to a point mutation in *M. morganii* gyrB (13), indicating resistance to fluoroquinolones. The accompanying plasmid contained genes conferring resistance to tetracycline (tet(A)_AJ517790) indicated by ResFinder.

## Data Availability

All sample reads can be under BioProject PRJNA1083987. Sequencing reads of *M. morganii* PS00513 can be found at SRA SRR28557218, BioSample SAMN40740907, and genome submissions CP151817.1–CP151818.1.
